# Thyroid Grafting

**Published:** 1890-10-11

**Authors:** 


### SURGERY.

### THYROID GRAFTING.

At a recent meeting of the *Académie* M. M. Bettencourt and Serrona read an account of an operation performed by them for the relief of myxœdema, which consisted of the grafting of the two halves of a sheep's thyroid into the atrophied gland of the patient. They stated that the number of red corpuscles increased until they reached the normal proportion, speech became regular, perspiration was restored, the œdema was less and the duration of the catamenia reduced from twenty to four days.

